# Psychiatric Morbidity and Mortality-Survival Among Homeless People: Evidence from a Two-Decade Portuguese 333 Patient Cohort

**DOI:** 10.1192/j.eurpsy.2026.10472

**Published:** 2026-07-31

**Authors:** J. Gama-Marques, J. Henriques-Calado

**Affiliations:** 1 Consulta de Esquizofrenia Resistente, Hospital Júlio de Matos, Unidade Local de Saúde São José, Centro Clínico Académico de Lisboa, Lisboa, Portugal; 2 Clínica Universitária de Psiquiatria e Psicologia Médica, Faculdade de Medicina, Universidade de Lisboa, Centro Académico de Medicina de Lisboa, Lisboa, Portugal; 3 Centro de Investigação em Ciência Psicológica (CICPSI), Faculdade de Psicologia, Universidade de Lisboa, Lisboa, Portugal

## Abstract

**Introduction:**

Homelessness represents a critical determinant of mental disorders, closely linked to psychiatric morbidity, substance use, and premature mortality. However, longitudinal evidence on homeless psychiatric populations in Southern Europe is still limited.

**Objectives:**

This study aimed to characterize the socio-demographic, clinical, and mortality/survival outcomes of homeless psychiatric patients, with an emphasis on diagnostic patterns associations over a 20-year follow-up.

**Methods:**

A retrospective analysis was conducted on 333 homeless psychiatric patients monitored over two decades, based on electronic clinical records. Data included socio-demographic information, ICD-10 psychiatric diagnoses at baseline and last contact, lifetime comorbidities, utilization of psychiatric services, and mortality outcomes. Mortality distribution and diagnostic associations were examined using descriptive, cox regression, and inferential analyses.

**Results:**

The cohort was predominantly male (87.7%), with a mean age of 47 years at baseline and 56 years at final contact. Mean psychiatric follow-up extended for nine years, with limited-service utilization (*M*=1.5 hospitalizations; *M*=51 inpatient days). Overall mortality reached 31.2%, with a mean age at death of 61 years, considerably lower than national life expectancy. Alcohol abuse was present in 57.7% of deceased patients. Diagnoses most strongly associated with mortality included alcohol-related disorders (ICD-10 F10; χ²=20.53, *p*<.001), organic psychosis (ICD-10 F06; χ²=14.38, *p*<.001), and unspecified psychosis (ICD-10 F29; χ²=8.47, *p*<.05). Intellectual disability (χ²=9.36, *p*<.01) and comorbid substance use were also significant predictors. Survival analyses highlighted alcohol use disorders and organic psychosis as major negative prognostic factors, whereas patients with long-standing psychosis and intellectual disability demonstrated greater variability in survival outcomes.

**Conclusions:**

This 20-year study reveals a substantial mortality burden among homeless psychiatric patients, strongly linked to alcohol-related disorders and organic psychosis. The findings underscore the critical need for integrated and diagnosis-specific interventions to mitigate premature mortality in this vulnerable homeless psychiatric population.

**Disclosure of Interest:**

None Declared

